# Eligibility determination for clinical trials: development of a case review process at a chiropractic research center

**DOI:** 10.1186/1745-6215-15-406

**Published:** 2014-10-24

**Authors:** Robert D Vining, Stacie A Salsbury, Katherine A Pohlman

**Affiliations:** Palmer College of Chiropractic, Palmer Center for Chiropractic Research, 741 Brady St., Davenport, IA 52803 USA; Department of Pediatrics Faculty of Medicine and Dentistry, 8B19 Edmonton General Hospital, University of Alberta – CARE Program, 11111 Jasper Avenue, Edmonton, AB T5K 0 L4 Canada

**Keywords:** Chiropractic, Complementary and alternative medicine, Clinical trials, Diagnosis, Eligibility determination, Low back pain, Mechanical, Neck pain, Patient recruitment, Patient safety, Patient selection, Randomized

## Abstract

**Background:**

Systematic procedures addressing the limitations of eligibility determination are needed to improve the quality of participant recruitment and enrollment in randomized clinical trials. This paper describes an eligibility determination process developed by and in use at a chiropractic research center engaged in community recruitment for clinical trials studying spinal pain conditions.

**Methods:**

A team of investigators developed a case review process for application across clinical trials involving chiropractic care. Study personnel representing key study roles including research clinicians, study coordinators, a project manager, and at least one investigator convene in person to determine eligibility for participants following baseline study visit examinations. The research clinician who performed the eligibility examination presents the case and a moderator leads the case review panel through a structured discussion including diagnosis, eligibility criteria, definition review, and clinical precautions. Panel members provide clinical recommendations and determine final eligibility using a structured and moderated voting process.

**Results:**

Through the case review process for three externally funded clinical trials for participants with neck and low back pain, we presented 697 cases, rendering 472 participants eligible for enrollment and excluding 225 individuals. The most common reasons for case review exclusions across the three trials included neck or back pain not meeting diagnostic classifications, safety concerns related to treatment or testing, referral for further evaluation or treatment, and compliance concerns.

**Conclusions:**

The case review process uses the expertise of study coordinators, research clinicians, project managers, and investigators to render eligibility decisions consistent with study aims for the duration of the trial. This formal eligibility determination process includes steps designed to mitigate the potential for participant misclassification from clinician advocacy or misunderstanding of eligibility criteria, and helps ensure that participants can safely take part in study procedures.

**Trial registration:**

The three trials discussed in this article were registered in ClinicalTrials.gov with the ID numbers of NCT00830596 (27 January 2009), NCT01312233 (04 March 2011), and NCT01765751 (30 May 2012).

## Background

Eligibility criteria for clinical trials are designed to ensure the safety of research participants [1] and reflect the scientific objectives of a study [2]. Automated eligibility determination systems help strengthen the enrollment process by standardizing decisions so that study personnel do not influence the selection process [3, 4]. However, in most clinical trials, investigators or their designees determine whether individual participants meet selection criteria, a process vulnerable to selection bias because of subjective interpretations [5] and the personal views of study personnel [6]. Thus, a challenge for clinical trial researchers is to render eligibility determination with procedures that facilitate consistent decision-making and avoid enrollment error, thereby reducing the opportunity for participant risk [1, 7, 8]. Determinations that lack a consistent and transparent process may lead to an unrepresentative sample [9] and influence the generalizability of study results [10, 11].

Although inclusion and exclusion criteria are reported routinely in scientific publications [12], the specific processes by which participant eligibility is determined and selection bias mitigated are often unclear. A 2010 systematic review studying interventions for improving adherence to eligibility criteria in randomized controlled trials did not find any primary publications describing such procedures [5]. Thus, there is a need to report eligibility determination procedures specifically designed to address potential limitations and to reduce the need for clinical trial managers to continually re-invent this process, which is necessary when established methods are not yet documented or disseminated broadly [13].

This article describes a multidisciplinary eligibility determination process developed and in use at a research center engaged in community-based recruitment for clinical trials of chiropractic care for non-surgical spinal pain. Our research teams developed this process to determine eligibility requiring clinical decision-making for three federally-funded randomized clinical trials in the United States: Trial 1 was a study of spinal manipulation with physiological and kinetic measurements for 221 adults aged 21 to 65 years with sub-acute or chronic low back pain [14]; Trial 2 was a study of collaborative care involving chiropractic and medical care for 131 participants with low back pain in adults aged over 64 years [15]; and Trial 3 was a study utilizing spinal traction mobilization for 48 adults aged 18 to 70 years with neck pain.

## Methods

### Ethics approval

Trials 1 and 3 were overseen by and received ethical approval from the Palmer College of Chiropractic Institutional Review Board (assurance numbers 2007M093 and 2012G151). Trial 2 was overseen and approved by both the Palmer College of Chiropractic and Genesis Health Systems Institutional Review Boards (assurance numbers 2011G138, 11-005). The institutional review boards approved the study protocols that included the case review process discussed in this manuscript. All study participants provided written informed consent.

### Clinical trials overview

We developed our case review process for eligibility determination during three separate clinical trials conducted with overlapping enrollment periods over a 5 year timespan. Trial 1 [14] was conducted to determine pre- to post-treatment changes in postural sway following spinal manipulation. Participants with acute, sub-acute, or chronic low back pain received 13 treatments over 6 weeks of either i) high velocity, low amplitude spinal manipulation; ii) low velocity, variable amplitude spinal manipulation; or iii) sham treatment. Sensorimotor measures included postural sway, repositioning accuracy, and response to a sudden load. Table 1 includes the specific eligibility criteria for Trial 1.

**Table 1:** **Eligibility criteria determined during case review for Trial 1**

**Inclusion criteria**	**Rationale**
Acute, sub-acute, or chronic low back pain matching Quebec Task Force classifications 1, 2, 3, or 7	Low back pain, uncomplicated by known nerve root compression, neurological signs, or prior surgery
**Exclusion criteria**	**Rationale**
Bleeding disorders	Potential intolerance to biomechanical testing or treatment protocols
Vascular claudication	Potential intolerance to biomechanical testing protocols
Bone and joint abnormality	Potential intolerance to biomechanical testing or treatment protocols
Inflammatory or destructive tissue changes to the spine	Potential intolerance to biomechanical testing or treatment protocols
Osteoporosis	Potential intolerance to biomechanical testing or treatment protocols
General poor health	Overall condition is too poor to tolerate treatment and biomechanical testing procedures
Neuromuscular diseases	Condition may interfere with data collection/interpretation
Peripheral neuropathies	Condition may interfere with data collection/interpretation
Prior spinal surgery	Condition may interfere with data collection/interpretation
Suspicion of drug or alcohol abuse	Condition may interfere with data collection, ability to comply with study protocol, and requires referral
Contraindication to spinal manipulation	Safety concern for treatment protocols
Low back pain matching Quebec Task Force classifications 4 to 6 and 8 to 11	Condition of sufficiently complicated nature to cause intolerance to biomechanical testing procedures or data collection
Cauda equina syndrome	Requires emergency surgical evaluation
Further diagnostic procedures other than dipstick urinalysis or X-rays	Advanced diagnostic tests outside study scope
Compliance concerns	May compromise ability to adhere to study protocol

Trial 2 [15] was a 12-week study conducted to evaluate the feasibility of a collaborative care model for chronic low back pain treatment in adults 65 years or older. Participants were allocated to one of three treatment groups: i) conventional care by a family medicine physician; ii) medical and chiropractic care separately but concurrently provided by a family medicine physician and a doctor of chiropractic; and iii) collaborative care by both a family medicine physician and a doctor of chiropractic. Table 2 lists the eligibility criteria for Trial 2 as listed in the study protocol.

**Table 2:** **Eligibility criteria determined during case review for Trial 2**

**Inclusion criteria**	**Rationale**
Low back pain diagnosis consistent with Quebec Task Force classifications 1 to 9	Low back pain classifications commonly treated by both doctors of chiropractic and primary care physicians
**Exclusion criteria**	**Rationale**
Low back pain diagnosis consistent with Quebec Task Force classification of 10 or 11	Classification 10 (chronic pain syndrome) and Classification 11 (other low back pain causes such as visceral disease, metastasis, and spondylitis) require treatment outside the scope of the trial
Spinal surgery in past 3 months	Potential to confound health outcomes
Fracture in any location in the body in 6 weeks	Recent fracture may influence ability to measure pain-related health outcomes; participant safety concern
Active carcinoma/metastatic disease or current treatment for any form of cancer	Serious health condition requiring medical treatment during study period; participant safety concern
Serious concomitant illness or co-morbidity preventing delivery of care of any available treatments	Potential to confound health outcomes
Serious concomitant illness or co-morbidity requiring coincident medical treatment	May interfere with study requirements or pose a significant scheduling burden to participants during study period
Contraindication to chiropractic care in primary treatment area	Safety precaution for treatment protocols
Aortic aneurysm (or suspicion of) >5 centimeters	Safety precaution for treatment protocols; need for referral for further evaluation or treatment
Need for advanced laboratory testing or diagnostic imaging or referral to a healthcare provider	Safety precaution for treatment protocols; potential need for further evaluation or treatment outside that provided by study protocol
Other clinical concerns for safety of available treatments	Safety precaution for treatment protocols
Activities of daily living, mobility, or sensory impairment	Severe impairments may pose a safety concern or impair delivery of available treatments in outpatient study facilities
Cognitive or memory impairment	May prohibit informed consent or compromise safety due to potentially reduced comprehension or compliance with study procedures
Suspicion of alcohol or drug abuse	May interfere with data collection, ability to comply with study protocol, and requires referral/management
Compliance concerns	May compromise ability to adhere to study protocol

Trial 3 was conducted to study a manual cervical distraction procedure for adults with radiating neck pain. Trial 3 represents the most recent iteration of the case review process and was conducted to i) measure the ability of chiropractic research clinicians to deliver a cervical traction mobilization procedure within specified force ranges and ii) provide preliminary outcomes data for a future full-scale clinical trial. There were five study visits and all included manual cervical distraction delivered within three treatment group-specific force ranges. Biomechanical procedures included repeated neck range of motion testing in multiple positions and performing repeated isometric muscular contractions in the cervical region. Table 3 presents eligibility criteria as listed in the study protocol and assessed during case review for Trial 3.

**Table 3:** **Eligibility criteria assessed during case review for Trial 3**

**Inclusion criteria**	**Rationale**
Neck or neck-related upper limb pain consistent with Quebec Task Force classifications 2 to 4	Neck pain radiating to proximal or distal extremity with or without neurological signs is commonly treated by doctors of chiropractic using study procedures
Naïve to flexion-distraction manual therapy procedures to cervical area	Requires participant unfamiliarity with study interventions
**Exclusion criteria**	**Rationale**
Neck pain consistent with Quebec Task Force classifications 1, 5 to 11	Classifications represent diagnoses that may require individualized treatment not available in trial; condition(s) may limit interpretation of study measurements
Bone/joint pathologies representing a contraindication to study procedures, including but not limited to:	
1) Inflammatory arthritis involving the cervical spine: i.e., rheumatoid arthritis, systemic lupus erythematosus, etc.	Study prescribed treatments are not intended for these conditions; may require referral and interfere with data collection and interpretation
2) Any condition representing neurological (spinal) instability in cervico-thoracic spine	Condition requires referral for evaluation or care outside study scope
3) Tumors, within or adjacent to the cervico-thoracic spinal canal	Condition requires evaluation or care outside study scope
4) Arnold Chiari malformation	May represent condition requiring referral for evaluation or care and limit interpretation of study measurements
5) Disorders known to exhibit spinal joint hypermobility, such as: Marfan syndrome, Ehlers-Danlos syndrome, osteogenesis imperfecta	May represent condition requiring referral; may interfere with data collection and interpretation
6) Cervico-thoracic disc herniation or prolapse demonstrating advancing neurologic deficits	Condition requires referral for evaluation or care outside study scope
7) Sequestered intervertebral disc fragment or loose bodies within the spinal canal, lateral recess, or intervertebral foramen	Safety precaution for available treatment protocols and may require referral
8) Any condition not listed above representing a contraindication to or a safety risk for study procedures	Safety precaution for treatment or biomechanical testing protocols and may require referral
Any single or multisegmental fusion (surgical or congenital) of the first through the seventh cervical vertebrae	Intervention hypothesizes that joint distraction and intervertebral disc pressure change are principal therapeutic mechanisms
Safety precaution (e.g., inability to safely ambulate within clinic, dizziness when arising or lying on treatment table, anxiety from study procedures)	Safety precaution and may interfere with participant’s ability to comply with study protocol
Unable to tolerate study procedures	Safety precaution for treatment or biomechanical testing procedures
Simultaneous management for condition compromising ability to deliver study treatment or assess health status	Safety precaution for treatment protocols and may present an undue scheduling burden
Suspicion of alcohol or drug abuse	May interfere with data collection, ability to comply with study protocol, and requires referral
Cognitive or memory impairment	May prohibit informed consent or compromise safety due to potentially reduced comprehension or compliance with study procedures
Referral for evaluation/management of other condition(s) or further testing required for neck pain diagnosis	Safety precaution and extensive or advanced diagnostic testing outside study scope
Compliance concerns*	May compromise ability to adhere to study protocol

*Compliance definition: Participants identified with compliance concerns (e.g., travel or transportation issues, conflicts with work or other routinely schedule activities, concerns with family or caregiving responsibilities, repeated scheduling issues during baseline evaluations, reluctance to receive treatment from study doctors only) will be discussed at Case Review and considered for exclusion on a case-by-case basis.

### Automated eligibility screening process

Eligibility determination is a multi-staged process that begins when an interested community-based volunteer contacts our research center via phone, direct mail post card, e-mail, or in person to initiate the screening process [16]. Research personnel provide the volunteer with basic information about the trial and conducts a computer-assisted telephone interview (phone screen) to assess preliminary eligibility. A phone screen-eligible participant is scheduled for an in-person baseline visit that includes an informed consent process conducted by a study coordinator. A consented participant then completes research questionnaires and an interview with the study coordinator to review select eligibility criteria. The study coordinator enters objective eligibility data that do not require clinical decision making (e.g., pain rating, body mass index, depression index) into a web-based participant tracking system programmed to generate automated decisions for specific eligibility criteria [17]. Participants who do not meet eligibility criteria at this point are excluded from the study and thanked for their interest and participation.

If eligible after the interview with the study coordinator, the participant receives a clinical evaluation from a research clinician who is a licensed doctor of chiropractic. The clinical evaluation includes a detailed health history review and focused examination of the spinal region under investigation. Additional clinical evaluation may occur when the health history, symptoms or examination findings merit further assessment to obtain information necessary to render clinically-related eligibility decisions or to determine whether referral to another provider is warranted. Clinicians may also use standard radiography when clinically appropriate [18]. Following the clinical evaluation, the research clinician completes an eligibility questionnaire on the web-based tracking system, entering only objective inclusion or exclusion criteria that require clinical evaluation, but which are not related to the diagnosis under study such as surgical history or the presence of a fracture. Participants may be excluded at this point by the research clinician. Participants who remain eligible following the baseline clinical evaluation move forward for formal eligibility determination at the case review meeting.

### Case review eligibility determination

The case review panel consists of 6 to 12 study personnel representing key roles and includes 2 to 4 research clinicians, 2 to 4 study coordinators, 1 clinical project manager, and the senior research clinician who leads the panel and is a trial investigator. Other personnel or study investigators may also be included in the panel depending on trial requirements. At least half of the study coordinators and research clinicians participating on the panel are graduate students enrolled in a master’s degree program in clinical research. All graduate students are degreed healthcare professionals (doctors of chiropractic); case review serves as a foundational training experience in the conduct of clinical trials for these students.

**Figure 1:**
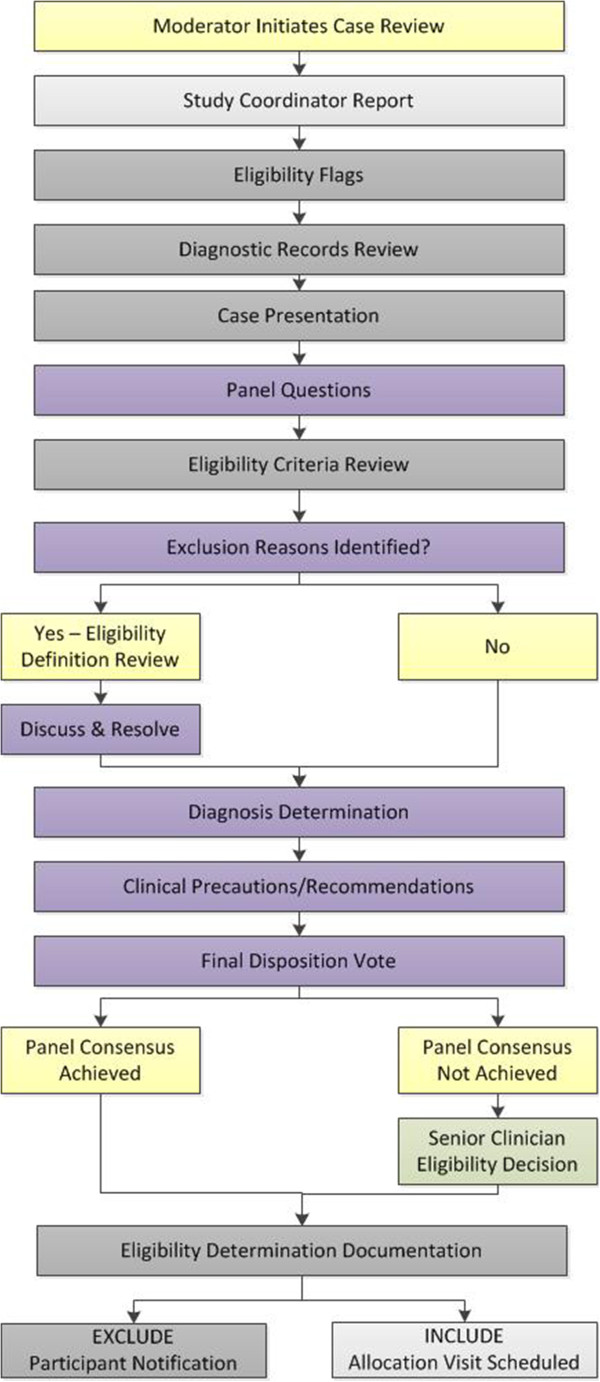
**Case review flowchart for the manual cervical distraction trial (Trial 3).** Yellow boxes, case review moderator process; Light grey boxes, study coordinator process; Dark grey boxes, research clinician process; Green boxes, senior clinician process; Purple boxes, case review panel process.

**Figure 2:**
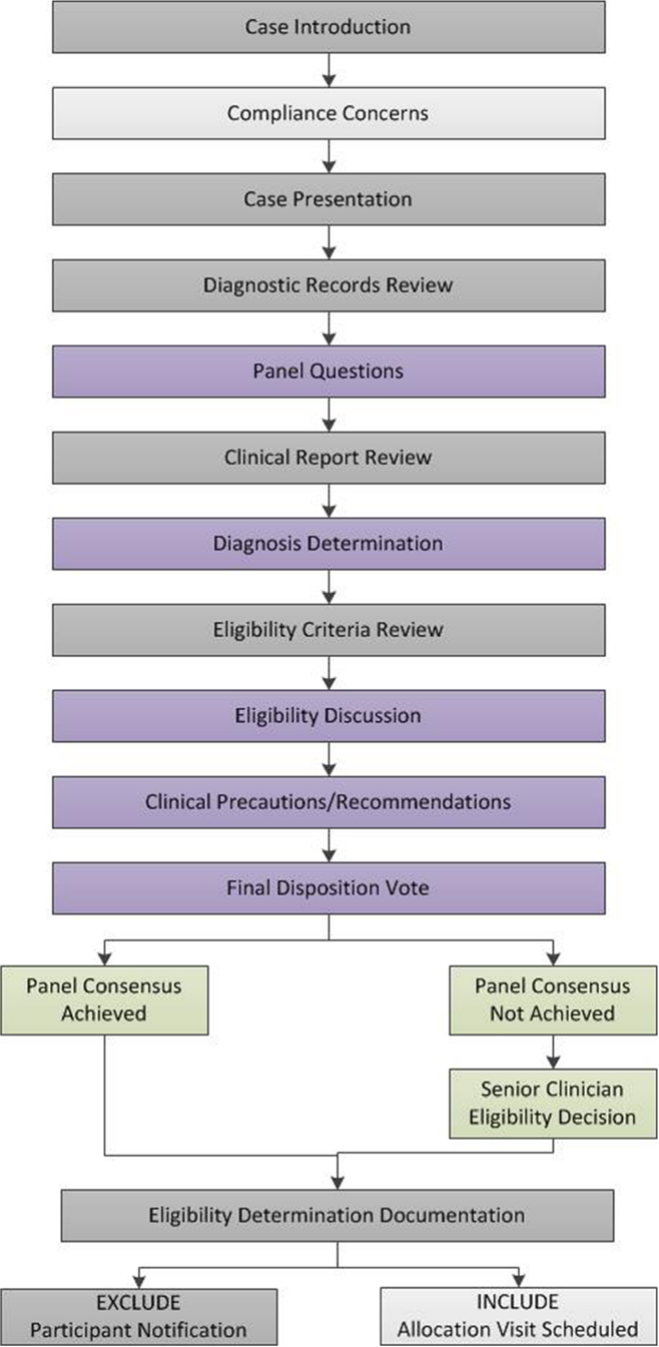
**Case review flowchart for the spinal manipulation and sensorimotor function trial (Trial 1).** Light grey boxes, study coordinator process; Dark grey boxes, research clinician process; Green boxes, senior clinician process; Purple boxes, case review panel process.

**Figure 3:**
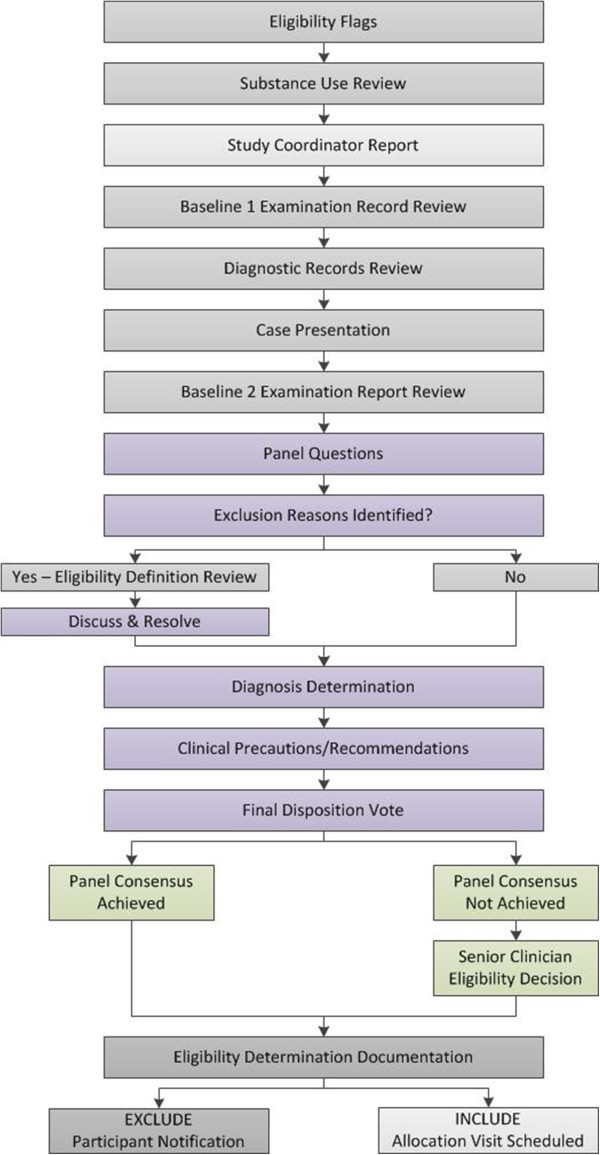
**Case review flowchart for the collaborative care trial (Trial 2).** Light grey boxes, study coordinator process; Dark grey boxes, research clinician process; Green boxes, senior clinician process; Purple boxes, case review panel process.

The case review panel typically meets twice per week for 1.5 hours per session. A study coordinator prepares paper and electronic records to assure their availability for case review meetings. Secure, password-protected network file servers hold documents containing personal health information. A web-based manual of operating procedures is also available to reference study protocols, eligibility criteria, and operational definitions for individual studies. The goals for the case review meeting are to: i) ensure complete and consistent case presentation; ii) encourage contributions by all panel members; iii) establish the clinical diagnosis [19]; and iv) reach consistent and scientifically-valid eligibility decisions.Case review is initiated by a study coordinator who also serves as moderator and leads the panel through the eligibility determination process using a stepwise flowchart. Figure 1 depicts the case review flowchart for Trial 3. Flowcharts depicting the case review process for Trial 1 and Trial 2 are illustrated in Figures 2 and 3, respectively. The moderator manages the case review discussion, ensures that each step of the process is addressed before subsequent discussion topics are introduced, and tallies consensus votes. Thus, the moderator allows the research clinician to focus on the presentation of the case, panel members to listen to the presentation and formulate discussion questions, and the project manager and senior clinician to oversee that the case review process addresses the eligibility criteria as outlined in the study protocol.

The moderator first asks the study coordinator who completed the informed consent process to introduce the participant and disclose information that could affect eligibility such as factors that could influence the ability to comply with study procedures (e.g., family responsibilities, work schedule, transportation issues). Next, the moderator directs the research clinician to present imaging findings and health records obtained elsewhere, when available. Following the health records review, the research clinician provides a formal case presentation including a detailed health history, focused history of the chief complaint, review of systems, and findings from the clinical evaluation.

Following the case presentation, the moderator designates each panel member an opportunity to ask questions. The research clinician answers panel members’ questions as they are posed. When all panel questions are addressed, the moderator directs the research clinician to review the study-specific eligibility criteria and their operational definitions, which are displayed electronically for the panel. The moderator then verbally polls each panel member individually regarding whether any particular exclusion criterion is met or requires discussion or clarification and tallies the poll results. If no exclusion concern exists, the moderator addresses the next item on the case review flowchart (e.g., diagnosis determination).

When an exclusion reason is posed by a panel member, the moderator reviews the corresponding study-specific eligibility definition. The moderator again verbally polls each panel member individually to determine the degree of consensus. Members provide verbal answers on their agreement with the exclusion criterion, along with justification for their opinion. When an eligibility decision can only be rendered after the diagnosis is determined, exclusion discussion is pended until after diagnosis confirmation by the panel. Each proposed exclusion decision occurs separately by a voting process. Consensus occurs when at least 80% of the panel members present agree with the eligibility determination decision. If consensus is not reached, the senior research clinician, or other designated trial investigator, renders final eligibility determination.

Once eligibility consensus is reached, the research clinician completes the participant-specific case review form located on a secure, password-protected web module programmed with study-specific exclusion criteria. The eligibility decision, along with the reasons for exclusion, comments regarding the case review process including whether or not consensus was reached, and the names of the research personnel who participated in the case review discussion are documented. The research clinician who performed the examination contacts participants excluded at case review to inform them of their eligibility status, report their findings, and provide clinical recommendations when appropriate. Referral recommendations for conditions such as abdominal aortic aneurysm, peripheral arterial disease, severe hypertension (urgency/crisis), evaluation for other suspected or unmanaged health conditions, and counselling for alcohol or drug dependence may also occur at this time. Participants included at case review are scheduled for the next study visit, which includes concealed allocation.

## Results

### Combined trial results

Our case review panel rendered eligibility determination decisions for three separate clinical trials from January 2009 through December 2013. The case review panel considered a total of 697 cases, excluded 225 participants, and allocated 400 participants across the three trials. Of the 225 exclusions at case review, 156 participants were excluded from Trial 1, 33 participants from Trial 2, and 36 participants from Trial 3. Table 4 outlines the eligibility determination dispositions across the three clinical trials. The number of participants excluded for each eligibility criterion are presented by study in Table 5 (Trial 1), Table 6 (Trial 2), and Table 7 (Trial 3).

**Table 4:** **Eligibility determinations for three clinical trials using the case review process**

Trial	Cases reviewed	Excluded at case review	Eligible at case review, did not enroll	Enrolled
Spinal manipulation and sensorimotor function trial (Trial 1)	428	156	51	221
Collaborative care trial (Trial 2)	173	33	9	131
Manual cervical distraction trial (Trial 3)	96	36	12	48
**Totals**	**697**	**225**	**72**	**400**

**Table 5:** **Exclusion decisions rendered during case review for Trial 1**

Exclusion criterion	Exclusion reasons for participants (n =122) who met a single exclusion criterion	Exclusion reasons for participants (n =63) who met multiple exclusion criteria	Number of participants meeting each exclusion criterion alone or combined with another
Other exclusions*	81	27	108
Safety risk^	17	14	26
Quebec Task Force for Spinal Disorders classification^#^	13	9	22
General poor health	4	10	14
Alcohol or drug use	3	3	6
Osteoporosis	1	2	3
Uncontrolled hypertension	2	0	2
Vascular claudication	1	1	2
Bleeding disorder	0	1	1
Prior spinal surgery	0	1	1

*Other exclusions included compliance concerns, referral needed, peripheral neuropathy, neuromuscular disease, or participant unable to tolerate study procedures.

^Safety risks related to treatment or testing procedures such as inflammatory spondyloarthropathy.

^#^Quebec Task Force for Spinal Disorders classifications 4 to 6 and 8 to 11 were exclusionary in this trial.

**Table 6:** **Exclusion decisions rendered during case review for Trial 2**

Exclusion criterion	Exclusion reasons for participants (n =22) who met a single exclusion criterion	Exclusion reasons for participants (n =11) who met multiple exclusion criteria	Number of participants meeting each exclusion criterion alone or combined with another
Evaluation or referral required*	7	9	16
Compliance^	7	5	12
Aortic aneurysm	2	1	3
Alcohol or drug use	2	1	3
Treatment safety	2	1	3
Concurrent clinical management^%^	1	2	3
Memory impairment	1	1	2
Quebec Task Force for Spinal Disorders classification^#^	0	1	1
Cancer, active treatment	0	1	1

*Evaluation or referral reasons such as recent medication changes with new onset of side effects, cardiovascular symptoms (e.g., unstable angina, new onset cardiac symptoms), progressive neurological signs/symptoms, depression or other mental health concern, etc.

^Compliance concerns such as transportation issues, family or work responsibilities, or reluctance for randomization to medical care group.

^%^Concurrent clinical management such as advanced renal disease or cardiovascular disease.

^#^Quebec Task Force for Spinal Disorders classifications 10 to 11 were exclusionary in this trial.

**Table 7:** **Exclusion decisions rendered during case review for Trial 3**

Exclusion criterion	Exclusion reasons for participants (n =25) who met a single exclusion criterion	Exclusion reasons for participants (n =11) who met multiple exclusion criteria	Number of participants meeting each exclusion criterion alone or combined with another
Quebec Task Force for Spinal Disorders classification – modified*	6	7	13
Unable to tolerate treatment or biomechanical test procedures at baseline visit	4	5	9
Referral for neck pain or other health condition	5	3	8
Alcohol or drug use	3	4	7
Safety	3	4	7
Compliance	2	1	3
Clinical management of another health condition required during study participation	1	2	3
Bone or joint pathology contraindicating study treatment	0	3	3
Not naïve to manual cervical distraction procedure	1	0	1

*Modified Quebec Task Force for Spinal Disorders classifications 1 and 5 to 11 were exclusionary for this trial.

Across the three trials, 72 participants who were deemed eligible for random allocation at case review were not randomized into a study, including 51 participants in Trial 1, 9 participants in Trial 2, and 12 participants in Trial 3. Of these, 28 participants (Trial 1 = 16 participants, Trial 2 = 7 participants, and Trial 3 = 3 participants), decided not to continue in their respective trial, usually citing time commitment conflicts with work or family as their reasons for non-participation. Another 20 participants in Trial 1 and 2 participants in Trial 3 did not return to the clinic after being eligible at case review, and thus were never randomly allocated to treatment. Finally, 24 participants (Trial 1 = 15 participants, Trial 2 = 2 participants, and Trial 3 = 7 participants) were excluded from their respective trials after being eligible at case review due to an exclusionary response at the pre-allocation interview, with the majority of these exclusions (n = 18) categorized as no longer meeting the established pain rating threshold for the study.

The most common reasons for exclusion across the three trials included neck or back pain not meeting the stipulated Quebec Task Force for Spinal Disorders classification [20]. Other common exclusions occurred for safety concerns related to treatment or testing procedures, referral for the neck or back pain condition or other health concern, participant-reported alcohol or drug use, and concerns related to a participant’s ability to comply with the study protocol. No participants were reported to the principal investigators as inappropriately enrolled in the respective trials. However, several participants withdrew from each trial and may, in some cases, represent persons who were judged incorrectly by the panel to be those who would be compliant with study protocols. In total, 34 participants withdrew or were lost to follow-up across the 3 trials: 22 participants from Trial 1; 9 participants in Trial 2; and 3 participants from Trial 3. We were unable to obtain reasons for withdrawal for the majority of these enrolled participants due to loss of contact.

### Trial 3 results

**Figure 4:**
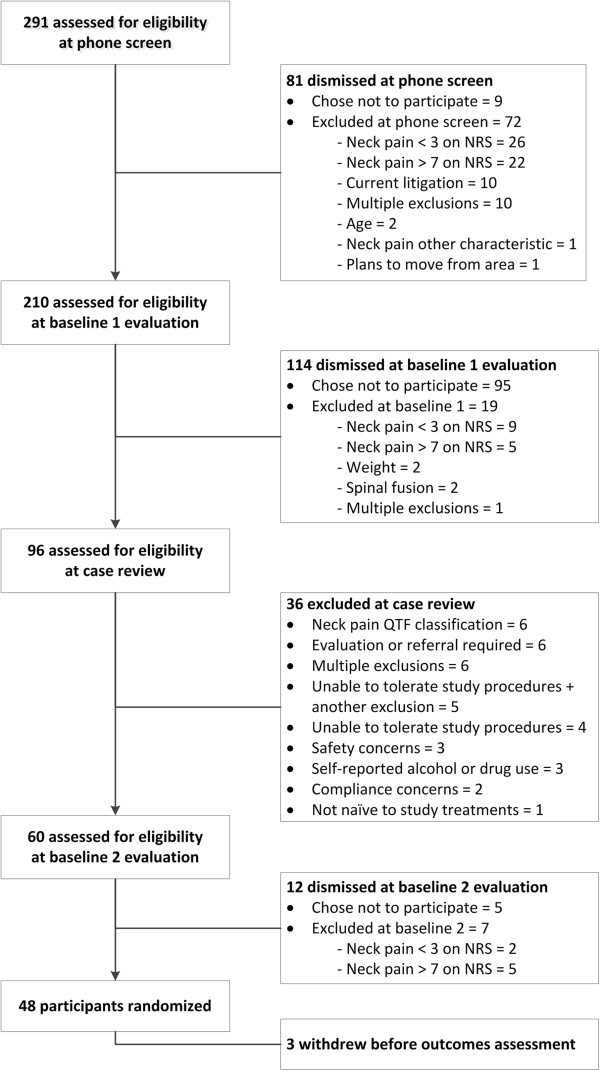
**Eligibility decisions from the multi-staged determination process for the manual cervical distraction trial (Trial 3).**

Figure 4 presents the eligibility decisions from Trial 3 as an exemplar of our multi-staged process for eligibility determination. Study personnel initiated 291 phone screens that 9 participants opted not to complete and through which 72 participants were excluded per the automated eligibility determination process, with the majority of these exclusions (n =48) being related to neck pain ratings outside those established for this study (3 to 7 on a 0 to 10 numerical rating scale). We scheduled 210 baseline 1 evaluations in which 95 participants opted not to continue in the study and 19 participants were excluded through the automated process, again, with most exclusions (n =14) related to self-reported neck pain rating. A total of 96 participants were brought forward to the case review meeting for eligibility determination, during which 36 were excluded from further participation. A total of 48 participants were allocated to treatment in Trial 3. Of the remaining 12 who were not allocated, 5 participants chose not to continue with the study and 7 rated their pain level outside the established inclusion criteria for the trial.

Table 7 presents the reasons why participants were excluded from Trial 3 at case review. All exclusions were due to a specific eligibility criterion. Twenty-five participants were excluded for a single exclusion criterion while 11 were excluded for multiple criteria (varying from 2 to 4 exclusions). Participants were most often excluded for neck pain not matching the modified Quebec Task Force for Spinal Pain classification ratings 2 to 4 [20, 21] (n =13), an inability to tolerate study procedures during the baseline examination (n =9), referral needed for neck pain or other health condition (n =8), self-reported high levels of alcohol or drug use on standardized substance use screening instruments (n =7) [22–24], and the inability to safely receive study treatments or perform biomechanical tests (n = 7).

We did not systematically collect the number of eligibility determinations where case review panel consensus was not reached until Trial 3. The study investigator made 3 of 96 (3%) decisions under circumstances where the case review panel did not achieve at least 80% consensus on the eligibility determination. The investigator excluded one participant on the suspicion of alcohol dependence or abuse, one for referral to evaluate another health condition, and one for the inability to safely tolerate the study treatment and biomechanical tests.

### Resource considerations

Eligibility discussions during case review varied from 15 minutes to 1.5 hours (averaging 30 minutes) per participant, meaning a substantial amount of combined personnel time is dedicated to the process. Lengthy decisions tended to occur early in the clinical trial, when participants were excluded for multiple reasons, or when new panel members, typically graduate students in the clinical research program, were present and more time was dedicated to reviewing study aims, definitions, protocols, and discussing rationale for decisions. At our facility, each panel member dedicated 7.5% of their working hours to case review meetings (3 hours/week).

## Discussion

The novel case review process presented here is a systematic method to mitigate selection bias in clinical trials of spinal conditions for subjectively determined eligibility criteria such as clinical diagnosis, tolerance of study procedures, participant safety, need for referral, substance abuse, and compliance concerns. Our structured case review process creates an environment for collaborative discussion, which includes vital contributions from clinicians, coordinators, and investigators who fill fundamentally different yet synergistic roles in the eligibility determination process for participants enrolling in a clinical trial. Each panel member is solicited to voice their perspective so as to capitalize on their unique expertise and experience to: i) facilitate clinical and scientific interpretation of eligibility criteria; ii) ensure participant safety regarding the ability to perform and receive trial procedures; and iii) mitigate selection bias by providing an environment to challenge, and therefore encourage, justifiable decision making, consistent with established eligibility definitions.

Unfortunately, we cannot compare the effectiveness or efficiency of our case review process with other methods for eligibility determination as the biomedical literature is silent regarding standard or systematic procedures designed to accurately enroll participants into clinical trials, particularly where clinical decision making is required [5]. This article represents a first step in describing systematic methods that specifically incorporate steps to mitigate selection bias where it is known to occur.

Our case review process is designed with the awareness that clinicians and other healthcare professionals do not always make eligibility determinations consistent with study aims or established inclusion and exclusion criteria. For example, clinicians may avoid enrolling eligible patients into a clinical trial because of the perceived effect such participation could have on the current doctor-patient relationship and to avoid feelings of responsibility if treatments were found to be unequal [25]. Clinicians also may prefer one trial therapy over another [6, 25–27] or avoid enrolling participants due to the concerns that the patient might receive a placebo [28]. Clinicians have reported reluctance to enroll study participants due to the complexity of clinical trials [26], the practical difficulty in following procedures, dislike of discussions with patients regarding uncertainty [25], and excessive time commitment [28]. Interviews with hospital personnel involved in one clinical trial found personal judgments about patients possibly explains why more than 50% of eligible patients did not receive an explanation of a study they could have been involved with [27].

Healthcare professionals may experience conflict between what they understand as their clinical responsibility to the patient and the variables by which trial eligibility decisions should be made. Clinicians may not fully understand key components of clinical research and may lack time, motivation, or opportunity to become knowledgeable about study procedures. However, skilled clinical assessment is usually essential for eligibility determination. To address this issue, our process includes clinicians and other study personnel to confirm interpretation of clinical information and to serve as a quality control mechanism. Our case review procedures and the inclusion of at least one study investigator [29] in the eligibility determination process are intended to prevent selection drift [30] and may be beneficial during trials in which participant recruitment lasts several years, occurs in multiple sites, or when implementation occurs with changing staff.

Eligibility assessment for clinical trials involving non-surgical spinal pain is challenging, in part because of the difficulties in establishing a definitive diagnosis [19, 31]. Other limitations include incongruent terminology among investigators, clinicians, and other study personnel with divergent areas of expertise involved in eligibility determination [32]. Without the use of consistent terminology it is easy to conceive of eligibility decisions that are inconsistent with criteria as envisioned by investigators. One example of inconsistent medical terminology is represented by the term lumbar spinal stenosis. Lumbar spinal stenosis is an imaging finding (a narrowed vertebral canal) whereas the clinical diagnosis of lumbar stenosis syndrome, with subtypes, is characterized by both imaging findings and distinctive symptoms. However, a variety of terms are used to describe the clinical condition [33, 34]. Without clear definitions of the clinical presentation, detailed examination procedures, and the specific factors indicating inclusion or exclusion, determining eligibility in a consistent manner can be difficult.

Because eligibility criteria can be unclearly defined [35] or interpreted inconsistently [27], we provide electronic access to study protocols and definitions during case review meetings. Real-time access to study protocols and definitions is designed to help prevent errant decisions due to complex study designs or protocols, and inadvertent misunderstanding [13]. We believe it is especially helpful when conducting simultaneous trials. We incorporate mandatory review of all eligibility criteria into our case presentations. To further prevent definition drift or systematic misunderstanding we include at least one investigator in case review meetings. The investigator also serves in the role of clarifying definitions, answering questions when they arise, and rendering final determination when consensus is not reached.

While our research center has automated the eligibility determination process for objective criteria, there remain eligibility decisions requiring clinical expertise that are more subjective in nature. The safety of participants when a manual therapy is delivered per the allocation scheme instead of tailored to an individual’s condition [36], the ability to tolerate and safely undergo challenging biomechanical tests without condition aggravation, the need to refer for further evaluation or treatment of a health condition, and the ability of a participant to comply with the study protocol [37] are eligibility considerations that might be swayed by misunderstanding or the personal biases of study personnel. The consensus-based case review process described here uses expertise from across the research team and a stepwise procedure to systematically render and support these decisions.

This eligibility determination process is not without its limitations. The diagnosis of spinal disorders is challenging [31, 38, 39]; participants often present with multiple overlapping conditions and a diagnosis is often based on the clinical interpretation of a wide range of information. We include multiple clinicians to aid this process. However, participants are not present during panel discussions of their case to provide clarifying information about their health history due to the challenges and costs of scheduling this additional visit. When the case review panel determines that additional information is needed, the eligibility decision is pended and the participant is re-contacted by a research clinician. Additional information is then presented to the panel at a future case review meeting for final eligibility determination. In addition, the act of verbally polling the opinions of panel members, necessary to confirm if reasons for individual eligibility decisions are consistent with study aims and operational definitions, can potentially hinder the free expression of thoughts and influence panel members.

This case review process is limited by personnel schedules and the amount of time and resources study teams allot to the process of eligibility determination. Our research center and personnel dedicate more time to the case review process than may be available elsewhere. We estimate that this case review process may work sufficiently with as few as three to four panel members. Further, key components of the case review process are transferrable to other research environments, including those that train students and clinicians to conduct clinical research [40]. We allocate extra time to case review at our research center to clarify procedures and to help educate graduate students serving as panel members on relevant topics such as clinical trial management [13], diagnostic reasoning, research ethics, and other critical aspects of clinical research. With experienced personnel and a developed process, the efficiency of case presentation and eligibility determination can be improved.

Exclusions during baseline visits conducted by a single study coordinator do not involve clinical decision making; rather, they are determined from objective measurements (e.g., weight, pain level). However, it is possible that enrollment error could occur due to incorrect data entry. While individual research clinicians also make eligibility determinations requiring clinical evaluation during the baseline process, these decisions are focused on facets of the participant’s health history that are similarly objective (e.g., surgical status in past year). Though procedures are designed to mitigate selection bias, subjective decisions are still made by personnel subject to unintentional bias and some decisions are made by a single individual when consensus is not reached.

## Conclusions

Case review is based on the concept of providing a formally structured decision-making process that includes at least one person from each of four roles: the consenting coordinator, examining clinician, project manager, and an investigator. It also includes designated steps to facilitate thorough case presentations, incorporate user-friendly and secure access to operational definitions for eligibility across multiple trials conducted simultaneously, moderate structured member discussions, and provide a formalized decision-making process to render eligibility determinations that reflect the scientific objectives of a clinical trial.

## References

[1] Macias WL, Vallet B, Bernard GR, Vincent JL, Laterre PF, Nelson DR, Derchak PA, Dhainaut JF (2004). Sources of variability on the estimate of treatment effect in the PROWESS trial: implications for the design and conduct of future studies in severe sepsis. Crit Care Med.

[2] Kasai T, Ohe Y, Nishio K, Kunitoh H, Tamura T, Sekine I, Kubota K, Yamamoto N, Nakamura Y, Shinkai T, Kodama T, Saijo N (1998). Factors that influence the eligibility of cases for inclusion in clinical trials. The Lung Cancer Chemotherapy Study Group of the Japan Clinical Oncology Group. Jpn J Clin Oncol.

[3] Fink E, Kokku PK, Nikiforou S, Hall LO, Goldgof DB, Krischer JP (2004). Selection of patients for clinical trials: an interactive web-based system. Artif Intell Med.

[4] Papaconstantinou C, Theocharous G, Mahadevan S (1998). An expert system for assigning patients into clinical trials based on Bayesian networks. J Med Syst.

[5] Simpson F, Sweetman EA, Doig GS (2010). A systematic review of techniques and interventions for improving adherence to inclusion and exclusion criteria during enrolment into randomised controlled trials. Trials.

[6] Donovan JL, Paramasivan S, De Salis I, Toerien M (2014). Clear obstacles and hidden challenges: understanding recruiter perspectives in six pragmatic randomised controlled trials. Trials.

[7] Umscheid CA, Margolis DJ, Grossman CE (2011). Key concepts of clinical trials: a narrative review. Postgrad Med.

[8] Department of Health and Human Services (2009). Code of Federal Regulations Title 45 Public Welfare, Part 46 Protection Of Human Subjects.

[9] Guyatt G, Rennie D, Meade MO, Cook DJ (2008). Users’ Guides to the Medical Literature: A Manual for Evidence-Based Clinical Practice.

[10] Van Spall HG, Toren A, Kiss A, Fowler RA (2007). Eligibility criteria of randomized controlled trials published in high-impact general medical journals: a systematic sampling review. JAMA.

[11] Fossa SD, Skovlund E (2002). Selection of patients may limit the generalizability of results from cancer trials. Acta Oncol.

[12] Schulz KF, Altman DG, Moher D (2010). CONSORT 2010 Statement: updated guidelines for reporting parallel group randomised trials. Trials.

[13] Farrell B, Kenyon S, Shakur H (2010). Managing clinical trials. Trials.

[14] Wilder DG, Vining RD, Pohlman KA, Meeker WC, Xia T, DeVocht JW, Gudavalli MR, Long CR, Owens EF, Goertz CM (2011). Effect of spinal manipulation on sensorimotor functions in back pain patients: study protocol for a randomised controlled trial. Trials.

[15] Goertz CM, Salsbury SA, Vining RD, Long CR, Andresen AA, Jones ME, Lyons KJ, Hondras MA, Killinger LZ, Wolinsky FD, Wallace RB (2013). Collaborative Care for Older Adults with low back pain by family medicine physicians and doctors of chiropractic (COCOA): study protocol for a randomized controlled trial. Trials.

[16] Hondras MA, Long CR, Haan AG, Spencer LB, Meeker WC (2008). Recruitment and enrollment for the simultaneous conduct of 2 randomized controlled trials for patients with subacute and chronic low back pain at a CAM research center. J Altern Complement Med.

[17] Pohlman K, Carber L, Vining R, Devlin T, Rice R, Salsbury S, Corber L, Hondras M, Long C, Goertz C (2012). Leveraging grant awards to enhance the research infrastructure at a CAM institution. BMC Complement Altern Med.

[18] Bussieres AE, Taylor JA, Peterson C (2008). Diagnostic imaging practice guidelines for musculoskeletal complaints in adults-an evidence-based approach-part 3: spinal disorders. J Manipulative Physiol Ther.

[19] Vining R, Potocki E, Seidman M, Morgenthal AP (2013). An evidence-based diagnostic classification system for low back pain. J Can Chiropr Assoc.

[20] Spitzer WO, LeBlanc FE, Dupuis M (1987). Scientific approach to the assessment and management of activity-related spinal disorders. A monograph for clinicians. Report of the Quebec Task Force on Spinal Disorders. Spine.

[21] Spitzer WO, Skovron ML, Salmi LR, Cassidy JD, Duranceau J, Suissa S, Zeiss E (1995). Scientific monograph of the Quebec Task Force on Whiplash-Associated Disorders: redefining “whiplash” and its management. Spine (Phila Pa 1976).

[22] Yudko E, Lozhkina O, Fouts A (2007). A comprehensive review of the psychometric properties of the Drug Abuse Screening Test. J Subst Abuse Treat.

[23] Gavin DR, Ross HE, Skinner HA (1989). Diagnostic validity of the drug abuse screening test in the assessment of DSM-III drug disorders. Br J Addict.

[24] U.S. Department of Health & Human Services, National Institutes of Health, National Institute on Alcohol Abuse and Alcoholism (2005). Helping Patients Who Drink Too Much, A Clinician’s Guide, Updated 2005.

[25] Taylor KM, Margolese RG, Soskolne CL (1984). Physicians’ reasons for not entering eligible patients in a randomized clinical trial of surgery for breast cancer. N Engl J Med.

[26] Mansour EG (1994). Barriers to clinical trials. Part III: knowledge and attitudes of health care providers. Cancer.

[27] Paramasivan S, Huddart R, Hall E, Lewis R, Birtle A, Donovan JL (2011). Key issues in recruitment to randomised controlled trials with very different interventions: a qualitative investigation of recruitment to the SPARE trial (CRUK/07/011). Trials.

[28] Benson AB, Pregler JP, Bean JA, Rademaker AW, Eshler B, Anderson K (1991). Oncologists’ reluctance to accrue patients onto clinical trials: an Illinois Cancer Center study. J Clin Oncol.

[29] Bell-Syer SE, Thorpe LN, Thomas K, Macpherson H (2011). GP participation and recruitment of patients to RCTs: lessons from trials of acupuncture and exercise for low back pain in primary care. Evid Based Complement Alternat Med.

[30] Tang H, Foster NR, Grothey A, Ansell SM, Goldberg RM, Sargent DJ (2010). Comparison of error rates in single-arm versus randomized phase II cancer clinical trials. J Clin Oncol.

[31] Waddell G (2005). Subgroups within “nonspecific” low back pain. J Rheumatol.

[32] Gennari JH, Sklar D, Silva J (2001). Cross-tool communication: from protocol authoring to eligibility determination. Proc AMIA Symp.

[33] Suri P, Rainville J, Kalichman L, Katz JN (2010). Does this older adult with lower extremity pain have the clinical syndrome of lumbar spinal stenosis?. JAMA.

[34] Katz JN, Harris MB (2008). Clinical practice. Lumbar spinal stenosis. N Engl J Med.

[35] Williams C, Hancock M, Ferreira P, Ferreira M, Maher C (2012). A literature review reveals that trials evaluating treatment of non-specific low back pain use inconsistent criteria to identify serious pathologies and nerve root involvement. J Man Manipulative Ther.

[36] Childs JD, Fritz JM, Flynn TW, Irrgang JJ, Johnson KK, Majkowski GR, Delitto A (2004). A clinical prediction rule to identify patients with low back pain most likely to benefit from spinal manipulation: a validation study. Ann Intern Med.

[37] Prisciandaro JJ, Rembold J, Brown DG, Brady KT, Tolliver BK (2011). Predictors of clinical trial dropout in individuals with co-occurring bipolar disorder and alcohol dependence. Drug Alcohol Depend.

[38] Wainner RS, Fritz JM, Irrgang JJ, Boninger ML, Delitto A, Allison S (2003). Reliability and diagnostic accuracy of the clinical examination and patient self-report measures for cervical radiculopathy. Spine (Phila Pa 1976).

[39] Kent P, Keating J (2004). Do primary-care clinicians think that nonspecific low back pain is one condition?. Spine (Phila Pa 1976).

[40] Sung NS, Crowley WF, Genel M (2003). Central challenges facing the national clinical research enterprise. JAMA.

